# Supplementary material for: The role of drop shape in impact and splash
Source: Nat Commun. 2021 May 24;12:3068. doi: 10.1038/s41467-021-23138-4 (PMC8144391; doi:10.1038/s41467-021-23138-4)
Supplement: Supplementary file 1 — Supplementary Information [file 41467_2021_23138_MOESM1_ESM.pdf]

Supplementary Information

The role of drop shape in impact and splash

Qingzhe Liu[1], Jack Hau Yung Lo[1*], Ye Li[2], Yuan Liu[1], Jinyu Zhao[1], Lei Xu[1*]

[1]Department of Physics, The Chinese University of Hong Kong, Hong Kong, China.

[2]CAS Key Laboratory of Quantitative Engineering Biology, Shenzhen Institute of Synthetic Biology, Shenzhen Institutes of Advanced Technology, Chinese Academy of Sciences, Shenzhen, China.

*e-mail: xuleixu@cuhk.edu.hk; hylo@cuhk.edu.hk

Supplementary Note 1 | Magnetic field strength and response time

To ensure that the magnetic field becomes negligible before drop impact, we measure the magnetic field versus time at the impact point with a Hall sensor, as shown below. The measurement shows that when the magnetic field is on, the field strength is 23.2 mT, and due to inductance the response time of turning off is about 15 ms. Therefore, we turn off the magnetic field at least 20ms before the drop impact happens, which guarantees that the magnetic field strength is negligible (smaller than 0.3 mT or 1.3% of the original value) during impact. Further measurements show that the liquid properties such as the surface tension and the viscosity remain unchanged under such a weak magnetic field, and thus the influence from magnetic field is negligible (see Supplementary Table 1 and 2 below).

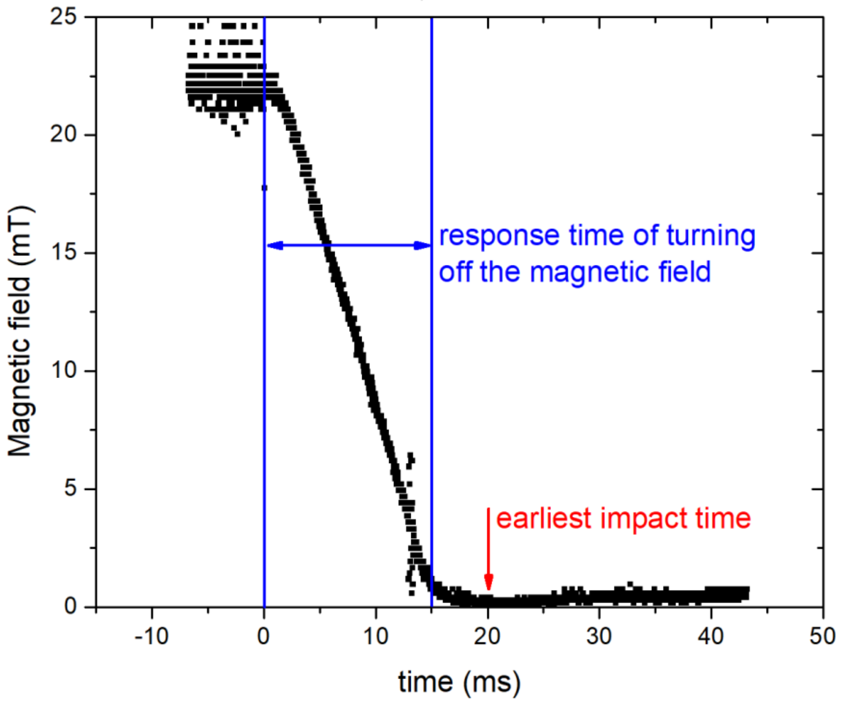

Supplementary Fig. 1 | Magnetic field strength at the impact position versus time. We turn off the magnetic field at least 20 ms before the drop impact happens, which guarantees that the magnetic field strength is negligible (smaller than 0.3 mT or 1.3% of the original value) during impact.

Supplementary Table 1 | Viscosity of the ferrofluid measured by rheometer under different magnetic field strength. The minimum field strength in the experiment region increases from 0 to 3.00 mT, which is 10 times stronger than the field during our impact process (smaller than 0.3 mT). However, no change in the viscosity is observed. Therefore, the weak residue of magnetic field in our experiment does not induce any observable change in viscosity.

Minimum magnetic field in the region (mT)	Maximum magnetic field in the region (mT)	Viscosity (mPa·s)
**0**	0	7.70 ± 0.01
**0.50**	0.77	7.69 ± 0.01
**2.03**	2.19	7.70 ± 0.01
**3.00**	3.25	7.70 ± 0.01

Supplementary Table 2 | Surface tension of the ferrofluid measured by pedant drop method under different magnetic field strength. The minimum field strength in the experiment region increases from 0 to 2.83 mT, which is over 9 times stronger than the field during our impact process (smaller than 0.3 mT). However, no change in the surface tension is observed. Therefore, the weak residue of magnetic field in our experiment does not induce any observable change in surface tension.

Minimum magnetic field in the region (mT)	Maximum magnetic field in the region (mT)	Surface Tension (mN/m)
**0**	0	18 ± 2
**0.11**	0.13	18 ± 2
**2.02**	2.39	18 ± 2
**2.83**	3.34	17 ± 2

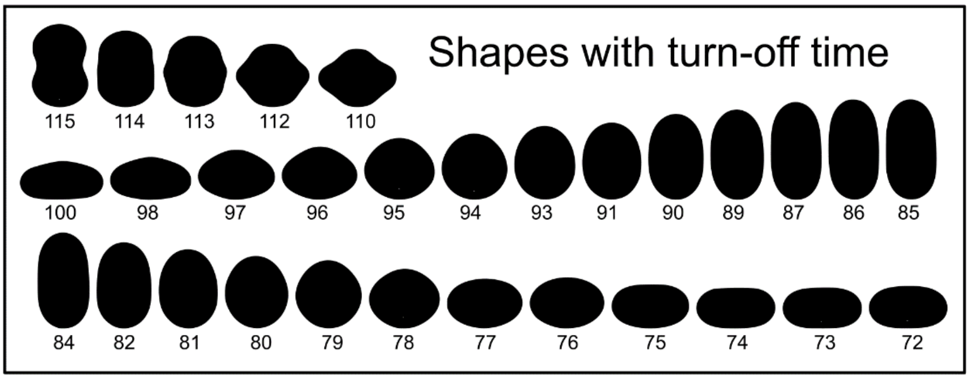

Supplementary Fig. 2 | Gallery of drop shapes with turn-off time of magnetic field. The number below each shape is the time interval in millisecond between the moment of drop detected by a laser trigger and the moment of turning off the magnetic field. By tuning this turn-off time, various shapes at the impact moment are produced, as shown in the gallery.

Supplementary Note 2 | Oscillation of drops

To demonstrate that drop oscillation does not influence the impact, we show the following calculation. The timescale of drop oscillation is $\tau = \pi\sqrt{\rho R^3/(2\sigma)} \approx 31$ ms calculated by Rayleigh oscillation frequency[1], where ρ is the liquid density, σ is the surface tension, and R is the drop radius. The characteristic time scale of the impact is defined as $T=L/2V$ with V the impact speed (2.9 ± 0.2 m/s), and L the drop length. In our study, L varies between 1.59 mm and 4.35 mm, and thus T ranges from 0.28 ms to 0.76 ms, which is much shorter than the oscillation period of 31 ms. Therefore, the drop shape can be considered as stable throughout the entire impact process.

In addition, we also measure the oscillation velocity, dL/dt, for each drop shape, as shown in Supplementary Fig. 3. Most shapes exhibit small oscillation velocities as shown by the orange region. To make sure that the oscillation speed does not affect the impact, we eliminate situations with large oscillation speeds (the green region), as represented by the shapes d and e. Only the shapes with small oscillation speeds are selected (the orange region) in our analysis.

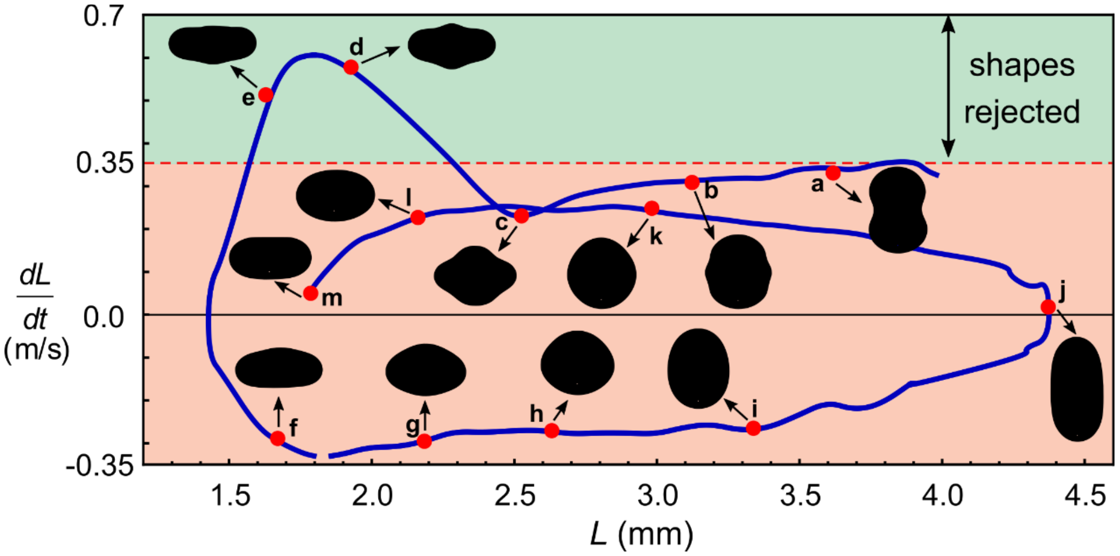

Supplementary Fig. 3 | The oscillation velocity of each drop shape. The blue curve represents all shapes we can achieve, shapes a to m (the red points) are selected as representative examples for demonstration. The drop shapes in green regions are rejected due to their high oscillation velocities, while the shapes in the orange region are selected due to their small oscillation velocities.

Supplementary Note 3 | Derivation of equation (3) and (4) of the main text

After a liquid drop impacts onto a solid substrate (see the drawing Supplementary Fig. 4 below), the liquid at the bottom part (region A) will be replaced by the substrate. We assume that this part of the liquid goes to region B and forms a cylinder[2–4]. By volume conservation, the volume of region A and B must be equal, or equivalently, volume of region A + C and B + C are equal, thus

$$\pi r^2 Vt = 2\pi \int_0^r x f(x) dx \tag{1}$$

where r is the radius of the wetting front, V is the impact velocity, t is the time after impact, and $f(x) = b - b\left(1 - x^n / a^n\right)^{1/n}$ is the drop shape described by the super-ellipse function (equivalent to equation (2) of the main text). The equation can be simplified to the dimensionless form,

$$t' = 1 - 2\int_0^1 u\left(1 - u^n r'^n\right)^{1/n} du \tag{2}$$

where $t' \equiv tV / b$ and $r' \equiv r / a$. Solving the equation, we get

$$t' = 1 - {}_2F_1\left(\frac{-1}{n}, \frac{2}{n}; \frac{n+2}{n}; r'^n\right) \tag{3}$$

with ${}_2F_1$ being the hypergeometric function. Expanding the equation in power series,

$$t' = \frac{2}{n(n+2)} r'^n + \frac{n-1}{2n^2(n+1)} r'^{2n} + \cdots \tag{4}$$

After neglecting higher order terms, we get $t' \approx \frac{2}{n(n+2)} r'^n$, which gives

$$r' \approx \left(\frac{n(n+2)}{2}\right)^{\frac{1}{n}} t'^{\frac{1}{n}} \tag{5}$$

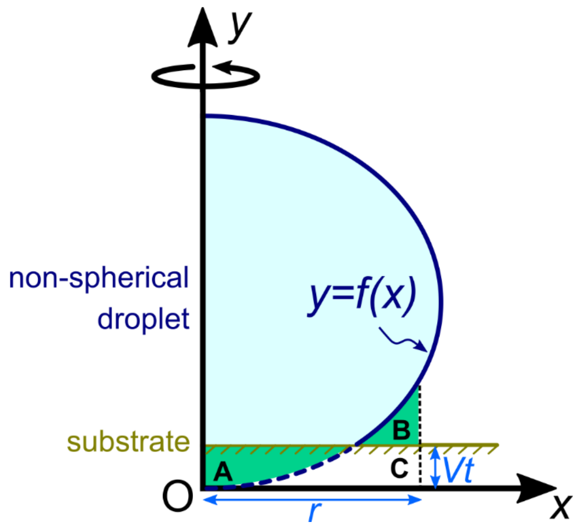

Supplementary Fig. 4 | A liquid drop impacting on a flat surface. By volume conservation, the volume of region A and B are equal. In the figure, r is the radius of the wetting front, V is the impact velocity, t is the time after impact, and $f(x)$ is the drop shape.

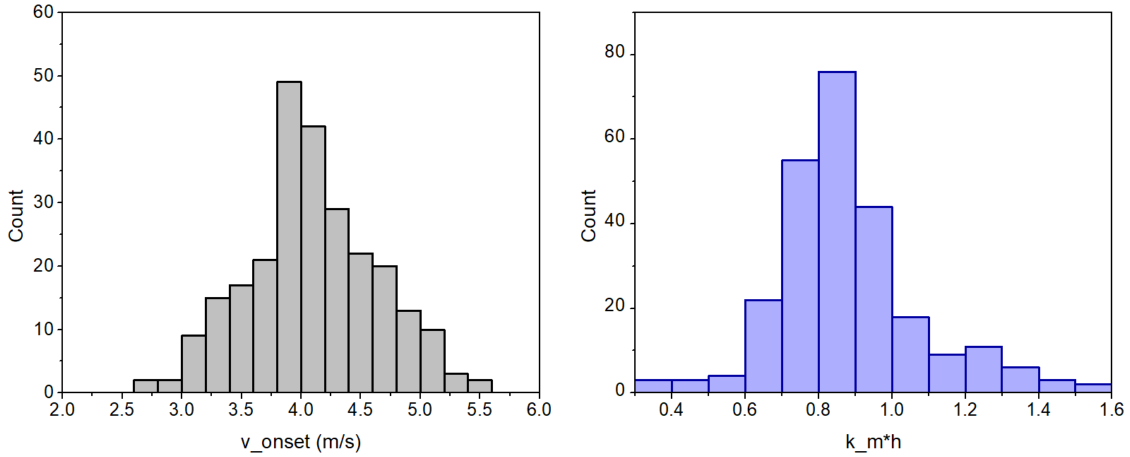

Supplementary Fig. 5 | Histograms of v_{onset} (left) and $k_m h$ (right) corresponding to Fig3d and 3e of the main text. The data in Fig. 3d and 3e are plotted in histograms as shown here. The total data points are N=256. The mean and standard deviation of v_{onset} is 4 m/s ±0.5 m/s (or ±13%), and the mean and standard deviation of $k_m h$ is 0.9 ±0.2 (or ±22%).

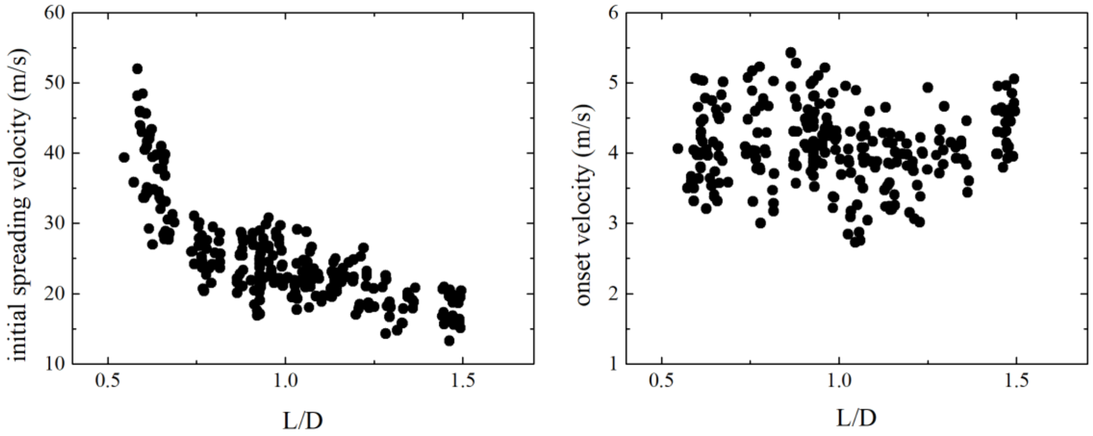

Supplementary Fig. 6 | Side-by-side comparison between the initial spreading velocities of all shapes measured at t=7.5 μs (left panel) and the onset velocities v_{onset} (right panel) for different shapes. As shown in the figure, the initial spreading velocities strongly depend on the drop shape, L/D; while the splash onset velocity, v_{onset}, exhibits a roughly constant trend with respect to the drop shape.

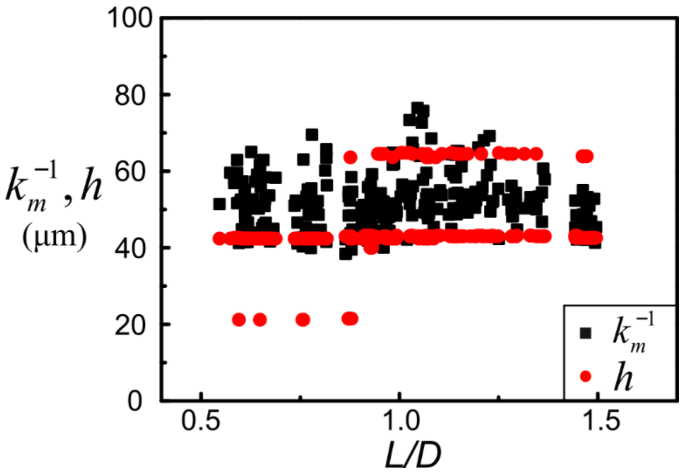

Supplementary Fig. 7 | Data of k_{m}^{-1} and *h*. The two length scales agree with each other as predicted by the splash criterion. Note that *h* is quite discrete due to the limited spatial resolution of our camera, which may explain some large deviations from unity in Fig. 3e in the main text.

Supplementary Note 4 | Derivation of viscous dissipation energy

The viscous dissipation energy of an axisymmetric flow is given by $E_{dis} = 2\mu\iint \phi dVdt$, where the viscous dissipation function is $\phi = \left(\frac{\partial u_r}{\partial r}\right)^2 + \left(\frac{u_r}{r}\right)^2 + \left(\frac{\partial u_z}{\partial z}\right)^2 + \frac{1}{2}\left(\frac{\partial u_r}{\partial z} + \frac{\partial u_z}{\partial r}\right)^2 \approx \frac{1}{2}\left(\frac{\partial u_r}{\partial z}\right)^2$ because of the thin thickness of the spreading liquid sheet. It is further approximated as $E_{\mathrm{dis}} \approx \mu\left(\frac{v_r}{h}\right)^2 \times V \times T$ where μ is the viscosity, v_r is the radial velocity, *h* is the thickness of liquid sheet, *V* is the volume and *T* is the timescale. Similar approximations can successfully deduce the scaling of maximum spreading diameter of drops by energy conservation[5–9]. In our model we take $V = \pi h R_{\mathrm{onset}}^2$, $T = R_{\mathrm{onset}} / v_{\mathrm{s}}$, and $v_r = v_{\mathrm{s}}$ where v_{s} is the spreading velocity of liquid sheet, *h* is the liquid film thickness, which is approximately the boundary layer thickness[10], and R_{onset} is the onset radius of splash. Therefore, we get $E_{\mathrm{dis}} \sim \frac{\mu v_{\mathrm{s}}}{h} R_{\mathrm{onset}}^3$.

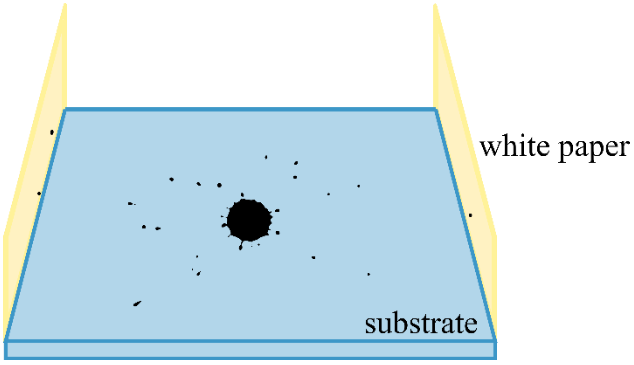

Supplementary Fig. 8 | Schematics of the setup of satellite droplets collection. The satellite droplets are collected by either the substrate or the white papers and leave black stains.

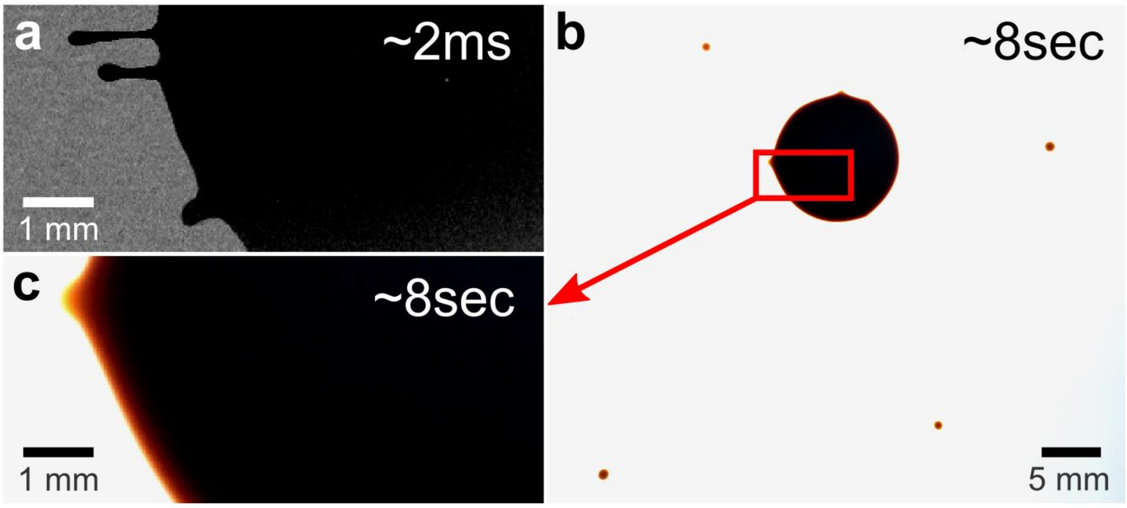

Supplementary Fig. 9 | Ejected satellite droplets get "swallowed" by the spreading parent drop. a, Ejected satellite droplets land in close proximity to the parent drop. The image is captured soon after impact (~2 ms) by high-speed camera. **b,** When we measure the total stain area, those ejected droplets have already been "swallowed" by the spreading parent drop. The image is captured at time ~8 s after impact. The red box indicates the same region shown in panel (**a**). **c,** The magnified image of the region inside the red box of panel (**b**).

Supplementary Note 5 | Experiments on different substrates

We performed our experiments on three different substrates. The first substrate, which is identical to that of the main text, is a glass microscope slide washed by acetone, IPA, and deionized water. The other two substrates are polymethyl methacrylate (PMMA or acrylic glass) surface and piranha-cleaned glass, which have lower and higher surface energy than the microscope slide respectively. The contact angles between a water drop and the acrylic glass, microscope slide, and piranha-cleaned glass are 74±4°, 26±4°, and 7±3° respectively. The corresponding literature values of the surface energy of the acrylic glass, microscope slide, and piranha-cleaned glass are 42 mN/m, 68 mN/m and 83 mN/m respectively[11,12]. The ferrofluid, whose surface tension is 19 mN/m, wets all three substrates.

The data of microscope slide (Black) is overlaid with the new data of acrylic glass (Pink) and piranha-cleaned glass (Green) in Supplementary Fig. 10 below, and they largely overlap. Therefore, our conclusion holds for substrates with different surface energy. This is also consistent with the previous study of Latka et al. that substrate wetting property has little or no effect on high-speed impact dynamics and splash[13].

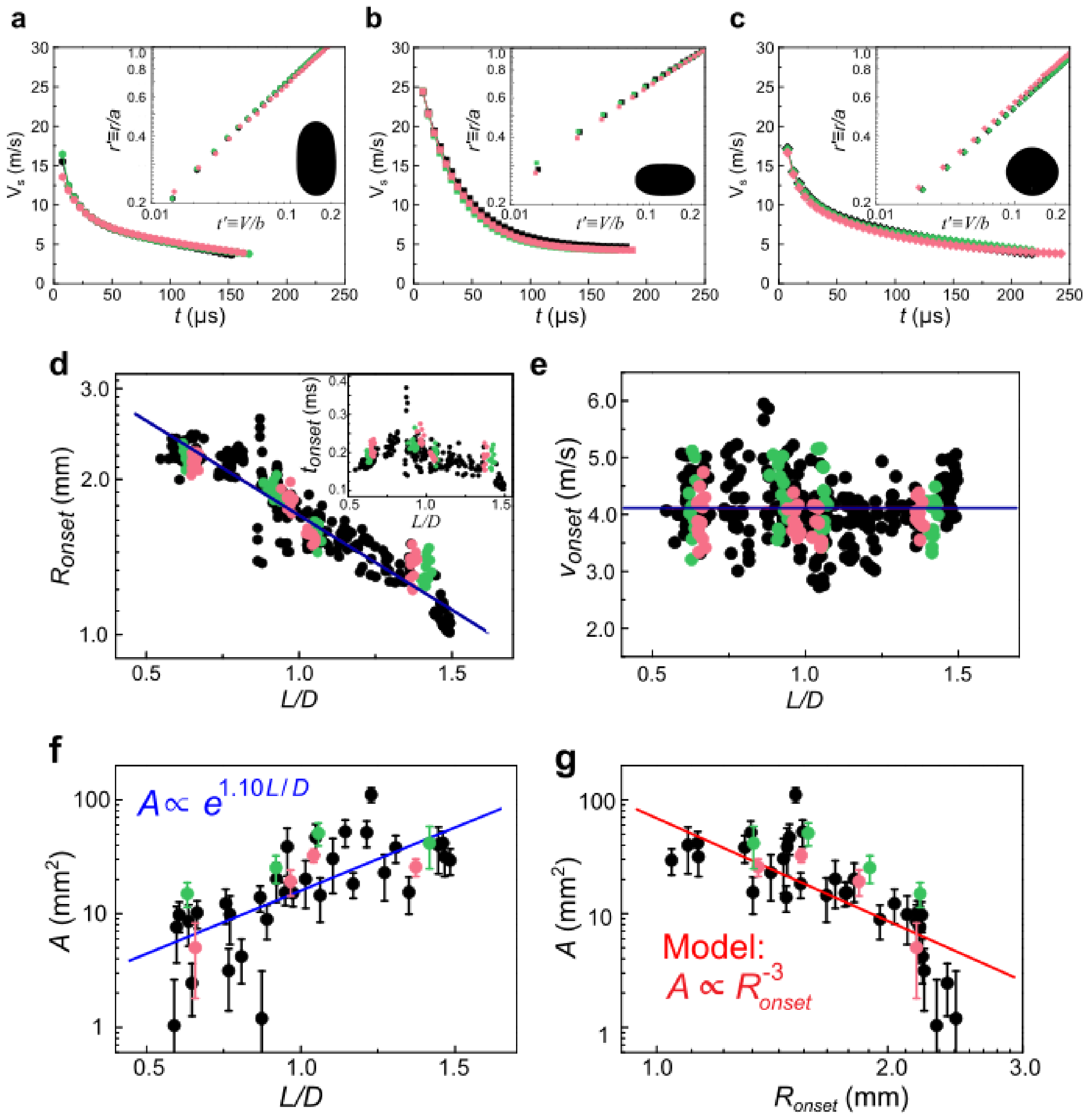

Supplementary Fig. 10 | The data of microscope slide (Black) are overlaid with the new data of acrylic glass (Pink) and piranha-cleaned glass (Green). The three sets of data overlap with each other. **a-c**, the drop spreading dynamics of three shapes on the three substrates overlap with each other. The main panels show the spreading velocity versus time and the insets show the spreading radius versus time. **d,** the splash onset location (main panel) and onset time (inset) on the three substrates agree with each other. **e,** splash onset velocities on three substrates overlap with each other. **f,** the splash amount on the three substrates overlaps with each other. **g,** the splash amount on the three substrates all agrees with the model. The error bars represent standard deviations.

Supplementary References

1. Becker, E., Hiller, W. J. & Kowalewski, T. A. Experimental and theoretical investigation of large-amplitude oscillations of liquid droplets. *J. Fluid Mech.* **231**, 189–210 (1991).
2. Oguz, H. N. & Prosperetti, A. Surface-tension effects in the contact of liquid surfaces. *J. Fluid Mech.* **203**, 149–171 (1989).
3. Kim, H.-Y., Feng, Z. C. & Chun, J.-H. Instability of a liquid jet emerging from a droplet upon collision with a solid surface. *Phys. Fluids* **12**, 531–541 (2000).
4. Zhang, L. V., Toole, J., Fezzaa, K. & Deegan, R. D. Evolution of the ejecta sheet from the impact of a drop with a deep pool. *J. Fluid Mech.* **690**, 5–15 (2012).
5. Chandra S. & Avedisian C. T. On the collision of a droplet with a solid surface. *Proc. R. Soc. Lond. Ser. Math. Phys. Sci.* **432**, 13–41 (1991).
6. Pasandideh-Fard, M., Qiao, Y. M., Chandra, S. & Mostaghimi, J. Capillary effects during droplet impact on a solid surface. *Phys. Fluids* **8**, 650–659 (1996).
7. Madejski, J. Solidification of droplets on a cold surface. *Int. J. Heat Mass Transf.* **19**, 1009–1013 (1976).
8. Clanet, C., Béguin, C., Richard, D. & Quéré, D. Maximal deformation of an impacting drop. *J. Fluid Mech.* **517**, 199–208 (2004).
9. Li, X.-H., Zhang, X.-X. & Chen, M. Estimation of viscous dissipation in nanodroplet impact and spreading. *Phys. Fluids* **27**, 052007 (2015).
10. Schroll, R. D., Josserand, C., Zaleski, S. & Zhang, W. W. Impact of a Viscous Liquid Drop. *Phys. Rev. Lett.* **104**, 034504 (2010).
11. Kowalski, A., Czech, Z. & Byczyński, Ł. How does the surface free energy influence the tack of acrylic pressure-sensitive adhesives (PSAs)? *J. Coat. Technol. Res.* **10**, 879–885 (2013).
12. Rhee, S. K. Surface energies of silicate glasses calculated from their wettability data. *J. Mater. Sci.* **12**, 823–824 (1977).
13. Latka, A., Boelens, A. M. P., Nagel, S. R. & de Pablo, J. J. Drop splashing is independent of substrate wetting. *Phys. Fluids* **30**, 022105 (2018).
