# Supplementary material for: The role of drop shape in impact and splash
Source: Nat Commun. 2021 May 24;12:3068. doi: 10.1038/s41467-021-23138-4 (PMC8144391; doi:10.1038/s41467-021-23138-4)
Supplement: Supplementary file 9 — Description of Additional Supplementary Files [file 41467_2021_23138_MOESM9_ESM.pdf]

Title: Supplementary Movie 1.
Description: A flat disc shaped drop (length L = 1.62 mm) impacts the substrate at V=3.08 m/s. The frame rate is 70, 000 fps and the entire process takes 6.9 ms.

Title: Supplementary Movie 2.
Description: A diamond shaped drop (length L = 2.43 mm) impacts the substrate at V=2.90 m/s. The frame rate is 70,000 fps and the entire process takes 4.2 ms.

Title: Supplementary Movie 3.
Description: A square shaped drop (length L = 2.54 mm) impacts the substrate at V=3.40 m/s. The frame rate is 50,400 fps and the entire process takes 5.8 ms.

Title: Supplementary Movie 4.
Description: A spherical drop (length L = 2.81 mm) impacts the substrate at V=2.72 m/s. The frame rate is 70,000 fps and the entire process takes 6.9 ms.

Title: Supplementary Movie 5.
Description: A peanut-like drop (length L = 3.96 mm) impacts the substrate at V=2.93 m/s. The frame rate is 70,000 fps and the entire process takes 6.9 ms.

Title: Supplementary Movie 6.
Description: An oval shaped drop (length L = 4.07 mm) impacts the substrate at V=2.75 m/s. The frame rate is 70,000 fps and the entire process takes 11.2 ms.

Title: Supplementary Movie 7.
Description: The impact dynamics of an elongated drop (length L = 4.33 mm), a flattened drop (length L = 1.51 mm) and a spherical drop (length L = 2.80 mm) on substrate recorded at the same frame rate 70,000 fps. They are all released from the height of 36.5 cm. The first three columns are recorded from the side and the last column presents the bottom view images of the third column. The entire process takes 5.3 ms.
